# Prevalence of attention-deficit hyperactivity disorder (ADHD): systematic review and meta-analysis

**DOI:** 10.1192/j.eurpsy.2024.1786

**Published:** 2024-10-09

**Authors:** Sara Popit, Klara Serod, Igor Locatelli, Matej Stuhec

**Affiliations:** 1 University of Ljubljana, Faculty of Pharmacy, Ljubljana, Slovenia; 2 Pomurske lekarne, Murska Sobota, Slovenia; 3 Department of Pharmacology, Faculty of Medicine Maribor, University of Maribor, Maribor, Slovenia; 4 Department of Clinical Pharmacy, Ormoz Psychiatric Hospital, Ormoz, Slovenia

**Keywords:** attention-deficit hyperactivity disorder (ADHD), epidemiology, prevalence, systematic review, meta-analysis

## Abstract

**Background:**

The estimates of attention-deficit hyperactivity disorder (ADHD) prevalence across various studies are significantly variable, contributing to uncertainty in ADHD prevalence estimation. Previous systematic reviews and meta-analyses have attributed this variability primarily to the methodological characteristics of the studies, including the diagnostic criteria, source of information, and impairment requirement for the diagnosis.

**Methods:**

Review identified studies reporting ADHD prevalence in representative samples of children and adults in Europe and worldwide. Studies that were conducted in the general population were included. We focused on studies that report ADHD prevalence based on clinical diagnosis (clinical diagnostic criteria based on the Diagnostic and Statistical Manual of Mental Disorders and International Classification of Diseases criteria, other diagnostic tools, such as various scales or interviews based on clinical diagnostic criteria). PubMed/Medline was searched to identify relevant articles published until 2024/2/01. The study was registered in PROSPERO (CRD42020200220) and followed Preferred Reporting Items for Systematic Reviews and Meta-Analyses 2020 guidelines for systematic review and meta-analysis.

**Results:**

In total, 117 studies were subjected to full evaluation. In the meta-analysis, 103 studies representing 159 independent datapoints were included. The overall prevalence of ADHD in register studies was 1.6%, 95% CI [0.9; 3.0], in survey studies 5.0%, 95% CI [2.9; 8.6], in one-stage clinical studies 4.2%, 95% CI [2.9; 6.0], and in two-stage clinical studies 4.8%, 95% CI [4.0; 5.8].

**Conclusions:**

Exact comparisons among studies with different diagnostic criteria and types of sampling can impact prevalence estimates. When comparing data from methodologically different studies, these factors need to be considered.

## Introduction

Psychiatric disease typically begins in childhood and adolescence, significantly impacting well-being and development [1, 2]. Childhood psychiatric problems are among the most common health issues, and their prevalence is increasing [2]. Attention-deficit hyperactivity disorder (ADHD) is one of the most widespread psychiatric diseases worldwide, especially in children and adolescents [3]. Psychiatric diseases often persist into adulthood, resulting in individual and collective social and economic burdens. This impact includes rising costs, education, welfare system, and administration of justice since it affects all aspects of life, such as school or work performance, relationships with family and friends, and community participation [1]. According to the study of Doshi et al., total annual costs in the United States have been estimated to range from US$143 billion to $266 billion, including health care and educational services for children and loss of income and productivity for adults [4, 5]. Basic National Health Service costs for ADHD (excluding medication costs) in England and Wales have been estimated at £23 million for the initial specialist assessment and £14 million annually for follow-up care [4, 6]. Shlander developed a model for ADHD drug costs in England and predicted in 2012 that costs would exceed £78 million [4, 7].

In 2022, Barican et al. [1] published a systematic review and meta-analysis that included 14 studies in 11 high-income countries with a pooled sample of 61,545 children aged 4–18 years. The most common psychiatric diseases were anxiety (prevalence 5.2%), attention-deficit/hyperactivity (3.7%), oppositional defiant (3.3%), substance use (2.3%), conduct (1.3%), and depressive (1.3%) disorders [1]. Cénat et al. [8] compared ADHD among children and adolescents of different races. They included 23 studies, and the pooled prevalence rate of ADHD was 15.9% with the pooled sample size of *n* = 218,445 [8]. Shooshtari et al. conducted an updated systematic review in 2022 in Iran, including 34 original studies covering 33,621 Iranian children, adolescents, and adults [9, 10]. The total prevalence of ADHD varies between 11% and 25.8% in preschool children, between 3.17% and 17.3% in school-aged children, and between 3.9% and 25.1% in adults [8]. Alhraiwil et al. [11] investigated the prevalence of ADHD in different age categories (children, adolescents, students) among Arab countries and reported variability across studies, with ADHD prevalence ranging between 0.46 and 19.6%. Ayano et al. [12] evaluated ADHD prevalence in children and adolescents in Africa, reporting a pooled prevalence of ADHD 7.47%. Liu et al. estimated the prevalence of ADHD among Chinese children and adolescents [13]. The prevalence estimates of ADHD in Mainland China, Hong Kong, and Taiwan were 6.5%, 6.4%, and 4.2%, respectively, with a pooled estimate of 6.3% [13]. These prevalence estimates align with a systematic review and meta-analysis from 2017, where an overall pooled prevalence of ADHD among children and adolescents in China was 6.26% (95% CI: 5.36–7.22%) [14]. Catalá-López et al. [15] investigated the prevalence of ADHD among children and adolescents in Spain, with the overall pooled prevalence estimated at 6.8%. Willcut [16] conducted a comprehensive meta-analysis on the prevalence of DSM-IV ADHD. While individual studies reported varied prevalence estimates, the pooled results suggested that the prevalence of DSM-IV ADHD was similar, whether ADHD was defined by parent ratings, teacher ratings, or a best estimate diagnostic procedure in children and adolescents (5.9–7.1%) or by self-report measures in young adults (5.0%) [16]. Thomas et al. [17] conducted a systematic review and meta-analysis to estimate ADHD prevalence in children, with an overall pooled estimate of 7.2%. Systematic reviews by Polanczyk et al. (2015; [3]) reported a worldwide ADHD prevalence in children and adolescents of 3.4% and found that estimates of ADHD prevalence were significantly variable (2014; [18]). In 2007, Polanczyk et al. published a systematic review and meta-regression analysis. The worldwide-pooled prevalence in subjects 18 years of age or younger from the general population or schools was 5.29%, and the prevalence estimate was associated with significant variability [19]. A few meta-analyses of epidemiological data on adult ADHD have been published; however, Simon et al. [20] published the first meta-analysis in 2009, estimating the prevalence of ADHD in adulthood at 2.5% (95% CI 2.1–3.1). Dobrosavljevic et al. researched ADHD prevalence in older adults. They concluded that pooled prevalence estimates differed significantly across assessment methods: 2.18% (95% CI = 1.51, 3.16) based on research diagnosis via validated scales, 0.23% (0.12, 0.43) relying on clinical ADHD diagnosis, and 0.09% (0.06, 0.15) based on ADHD treatment rates [21]. Song et al. [22] assessed the global prevalence of adult ADHD in the general population through a systematic review and meta-analysis. The prevalence of persistent adult ADHD was 2.58%, and that of symptomatic adult ADHD was 6.76% [22].

Worldwide, several systematic reviews and meta-analyses have been conducted to summarize the ADHD prevalence and analyze variation of prevalence estimates reported by individual studies. The variability in ADHD prevalence rates was mainly associated with the methodological characteristics of the studies, such as diagnostic criteria, source of information, and the impairment requirement for the diagnosis [18, 19, 23]. Geographical location was not a primary factor associated with the variability of prevalence estimates [16, 18, 19]. Variations in prevalence were also confirmed in the World Mental Health Surveys, where adult ADHD prevalence averaged 2.8% across surveys and was higher in high (3.6%)- and upper-middle (3.0%)- than low-/lower-middle (1.4%)-income countries [22].

Accurate prevalence estimates of psychiatric diseases in children and adolescents are crucial to evaluate and properly address burdens, as these disorders often persist into adulthood and can lead to the loss of human potential [1, 3]. Policymakers require prevalence data from multiple high-quality epidemiological studies using current or recent diagnostic standards with rigorous diagnostic measures [1]. Systematic review and meta-analysis methods are optimal choices for providing high-quality, relevant, accessible, and up-to-date information that is crucial for policymakers [1, 24]. In the context of the previous limitation in the meta-analyses and systematic reviews mentioned above and the high ADHD disease burden worldwide, the primary rationale for this review is to obtain the latest data on ADHD prevalence and differences in prevalence among different study types. These data would help calculate the disease burden and help policymakers and clinicians better plan resources for ADHD management.

## Methods

We followed the guidelines of the Preferred Reporting Items for Systematic Reviews and Meta-Analyses (PRISMA) Statement [25]. The protocol for this systematic review was registered in PROSPERO (CRD42020200220).

### Literature search

We conducted a search on PubMed/MEDLINE until 2024/1/02 for articles in all languages using search terms related to “Attention-Deficit/Hyperactivity Disorder” and “Epidemiology,” “Prevalence,” and “Point Estimate.” The detailed search strategy is available in the Supplementary material. No restrictions were applied concerning language or type of document; we included full-text published articles or conference proceedings.

### Selection criteria

We included observational cohort studies (retrospective, prospective, register-based population studies); cross-sectional studies; and clinical studies with participants from the general population (e.g., community samples, population-based registries, etc.) diagnosed with ADHD using either: a) clinical diagnosis according to the International Classification of Diseases (ICD) or DSM criteria reported in registers/medical files or self-reported medical history; or b) research diagnosis of ADHD, that is, meeting the threshold/cut-off levels on an ADHD-validated scales based on the DSM or ICD. We considered studies with data on ADHD prevalence in those populations.

We excluded articles that were not in English (515 articles). Additionally, we excluded studies conducted in nonrepresentative samples of the general population (e.g., only boys/men, siblings) or in selected areas (e.g., only rural/urban areas with small samples). Studies not focusing on the prevalence of ADHD per se, despite providing some data about it, as well as those presenting the lifetime prevalence of ADHD and including only patients with ADHD receiving pharmacological treatment, were also excluded.

### Data extraction

References to studies identified in electronic searches were managed in MS Excel®. Titles/abstracts were screened by two authors (SP, KŠ), and full-text articles were independently screened by two authors ( SP and KŠ). Senior authors (MŠ, IL) were consulted to reach a consensus when needed. Two authors ( SP and KŠ) independently extracted data. The following data were extracted: first author and year of publication; year of data collection (if applicable); country; age category; number of individuals with ADHD; sample size; and prevalence assessment method group (one-stage clinical studies, two-stage clinical studies, surveys, and registry studies on medical records data). Age categories were defined as preschool children (below 6 years), school children (6–12 years), adolescents (12–18 years), and adults (above 18 years). Combinations of age categories were also included. A margin of 6 years was allowed for the preschool and school children categories, while an age interval of 11–13 years was allowed for the categories school children and adolescents. For the adult category, the age range had to be over two decades. Prevalence estimates from the same study based on different countries, age groups, or time frames were considered separate datasets. In case of overlapping study samples, the study that was published earlier and/or the study that was the most pertinent to our criteria was included. If the prevalence estimate was not reported or could not be calculated based on data from the paper, a study was excluded from the systematic review.

### Data synthesis

Data synthesis was performed separately for each of the four prevalence assessment method groups. As a rule, three or more studies should be available for each subgroup. The meta-analysis of prevalence data and forest plot construction was performed in an R statistical environment. Age categories were used as subgroups in the meta-analysis (version 4.3.1), utilizing the function metaprop within the “meta” package. Due to the expected high heterogeneity, the random-effect model was applied and the restricted maximum-likelihood estimator was used for calculating between-study variance. The overall prevalence was calculated using the logit transformation. The confidence interval of the overall prevalence estimate was calculated based on Hartung-Knapp adjustment. Confidence intervals of prevalence for individual studies were calculated based on exact binominal intervals.

## Results

**Figure 1.**
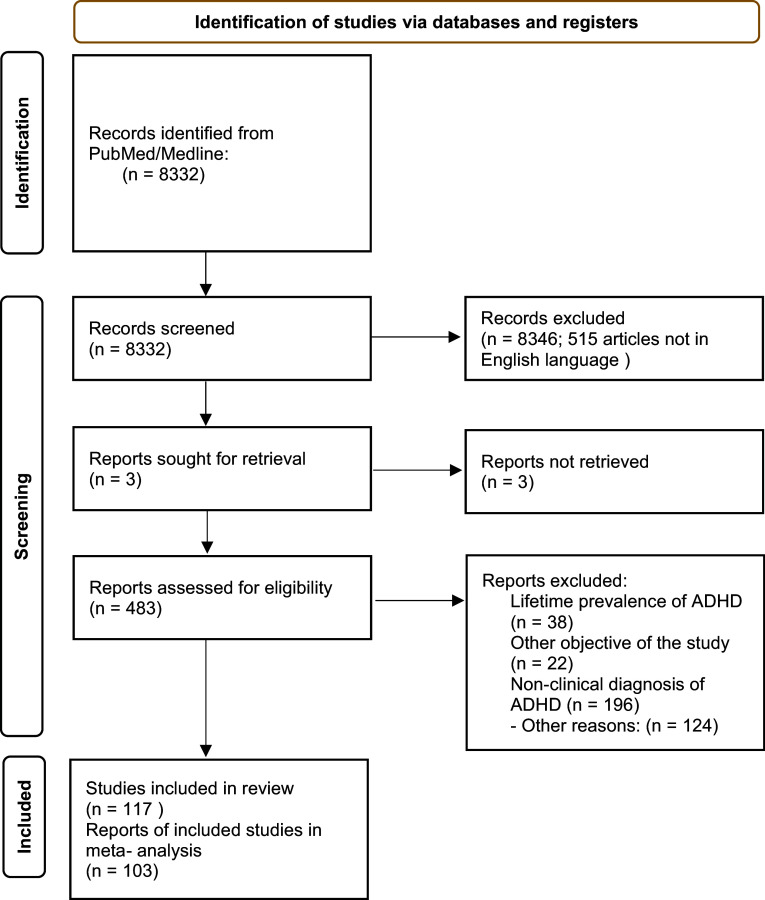
PRISMA flowchart.

In total, 8,332 records were retrieved and 508 studies were subjected to full evaluation. A total of 117 studies covering children, adolescents, and adults were included in the systematic review (Figure 1). Then, 103 studies representing 159 independent samples were included in the meta-analysis. The majority of these studies (116 studies) were conducted in different countries worldwide, while one study was conducted in 10 countries in the Americas, Europe, and the Middle East [23]. Of the 117 included studies, 26 studies were conducted in Asia (6 in China, 7 in India, 4 in Turkey); 32 in Europe, 26 studies in North America (19 of them in the United States); 3 studies in South America; 10 studies in Africa; 12 in the Middle East; and 3 in Oceania.

We summarized descriptive data for studies included in the systematic review in Tables 1–3.

**Table 1.** Descriptive data for studies included in the systematic review – register studies and surveys

First author and year of publication	Study population category	Sample size	Age range	Country	Region/city	Diagnostic standard	Diagnostic measure	Informant(s)	Timeframe	Type of study
Akmatov, 2018 [26]	Children and adolescents	6,007,414	5–14	Germany	Nationwide	ICD-10-GM	Electronic health records	Non explicit (physicians, pediatricians, specialists)	2009–2016	Register
Bachmann, 2017 [27]	Children, adolescents and adults	~24 million	0–17 18–69	Germany	Nationwide	ICD-10	Electronic health records	(Child and adolescent) psychiatrists, physicians, specialists	2009–2014	Register
Bannett, 2020 [28]	Children and adolescents	40,323	4–17	California	San Francisco bay area	ICD-10	Electronic health records	Clinicians	2015–2017	Register
Brook, 2005 [29]	Adolescents	543	15,5–17,3	Israel	Holon	DSM-4	Electronic health records	Teachers, psychologists or physicians	Non explicit	Register
Brownell, 2001 [30]	Preschool children, children, adolescents	314,153	0–19	Canada	Province of Manitoba	ICD-9-CM	Electronic health records	Physicians; psychiatrists; pediatricians; General Practitioners	April 1, 1995–March 31, 1996	Register
Burd, 2003 [31]	Preschool children, children, adolescents, young adults	129,138	0–21	United States	North Dakota	ICD-9	Electronic health records	Primarily physicians	1996–1997	Register
Butt, 2023 [32]	Preschool children, children, adolescents, young adults	49,031	1–24	Canada	Ontario	ICD-9, ICD-10-CA	Electronic health records	Family physicians	2016	Register
Chung, 2019 [33]	Children, adults	5,282,877 adults, 867 453 children	5–≥65	United States	Northern California	ICD-9, ICD-10	Electronic health records	Licensed psychiatrist, psychologist, or psychiatric nurse practitioner	January 1, 2007–December 31, 2016	Register
Davidovitch, 2017 [34]	Children and adolescents	138,725	5–17	Israel	Nationwide	DSM	Electronic health records	Neurologist/psychiatrist/qualified pediatricians	2005–2014	Register
Davis, 2019 [35]	Children	208,585	0–6	United States	Kentucky	ICD-9, ICD-10	Electronic health records	Psychiatrists; primary care physicians; neurologists	2012–2016	Register
Davis, 2021 [36]	Children and adolescents	304,951	6–17	United States	Kentucky	ICD-10	Electronic health records	Psychiatrists	2017	Register
Hauck, 2017 [37]	Preschool children, children, adolescents, adults	10,000	1–24	Canada	Ontario	ICD-10	Electronic health records	Medical expert (consultations with pediatricians, psychiatrists, psychologists and social workers)	December 31, 2011 (data extraction)	Register
Fast, 2024 [38]	Children	2,658	12	Sweden	Halland region	DSM–5	Electronic health records	Not explicit (physicians)	until 31st December 2019	Register
Giacobini, 2018 [39]	Adults	Annual population in Sweden	19–23,7	Sweden	Nationwide	ICD-10	Electronic health records	Clinician	2006–2011	Register
Giacobini, 2023 [40]	Preschool children, children, adolescents, adults	Total number of patients with population statistics for Sweden per year and region	0–17 ≥18	Sweden	Nationwide	ICD-10	Electronic health records	Specialist care physician	January 1, 2018–December 31, 2020	Register
Habdank-Kolaczkowski, 2023 [41]	Preschool children, children, adolescents	Nationally recognized and authoritative repository for health–related data	3–17	United States	Nationwide	DSM–5	Electronic health records	Non explicit (physicians)	1997–2018	Register
Hauck, 2017 [37]	Preschool children, children, adolescents, young adults	29,256 (10,000 randomly selected)	1–24	Canada	Ontario	ICD-10	Electronic health records	Family doctor, active/practicing physician, trained abstractors, a medical expert	December 31, 2011	Register
Holden, 2013 [42]	Children, adolescents and adults	143 million person–years of computerized data	6 to 17–adults	United Kingdom	Nationwide	Non explicit	Electronic health records	Specialist (Under NICE guidelines, diagnosis should be made by a mental health specialist	1998–2010	Register
Hong, 2014 [43]	Children and adolescents	8,218,252 (mean total)	6–18	South Korea	Nationwide	ICD-10	Electronic health records	Clinicians (psychiatry, pediatrics, and others)	January 1, 2007– December 31, 2011	Register
Knight, 2014 [44]	Adults	Approximately 3.5 million	18–100	United States	Southern California	ICD-9 CM	Electronic health records	Medical doctor	January 1st 2006–December 31st 2009	Register
Leache, 2021 [45]	Children and adolescents	124,582	5–19	Spain	Navarre	ICD-10	Electronic health records	General practitioner, pediatrician, specialist	2003–2019	Register
Leung, 2019 [46]	Preschool children, children and adolescents	Approximately 4.2 million	0–18	Canada	Alberta	ICD-9, ICD-10	Electronic health records	Psychiatrist	2008–2015	Register
McKechnie, 2023 [47]	Adults and children	7,655,931	3–99	United Kingdom	Nationwide	Read code system	Electronic health records	Primary care clinicians; specialists; specialist psychiatrist, pediatrician or other appropriately qualified healthcare professional with training and expertise in the diagnosis of ADHD	2000–2018	Register
Pérez–Crespo, 2020 [48]	Preschool children and children	1,114,226	4–17	Spain	Catalonia	ICD-9	Electronic health records	Non explicit	2009–2017	Register
Polyzoi, 2018 [49]	Adults	44,364	18–/	Sweden	Nationwide	ICD-10; DSM-5	Electronic health records	Treating medical doctor	January 1, 2006 through December 31, 2011	Register
Ramos–Quiroga, 2023 [50]	Children, adolescents and adults	1,000,000	1–>30	Spain	Nationwide	ICD-9	Electronic health records	Psychiatrists; medical professionals	2013–2018	Register
Riedel, 2021 [51]	Children and adolescents	2,156,733	3–17	Germany	Nationwide	ICD-10-GM	Electronic health records	Specialized physicians; psychiatrists	2009–2017	Register
Schlander, 2007 [7]	Children, adolescents and adults	2,238 million	Children–adults age 20 years and higher	Germany	Nordbaden	ICD-10	Electronic health records	Physician	Q1–Q4/2003	Register
Seo, 2022[52]	Preschool children, children and adolescents	Approximately 50 million	1–≥ 31	Korea	Nationwide	ICD-10	Electronic health records	Psychiatrists	January 1, 2008–December 31, 2018	Register
Štuhec, 2015 [53]	Preschool children, children and adolescents	394681 (2012)	0–19	Slovenia	Nationwide	ICD-10	Electronic health records	Psychiatrist	1997–2012	Register
Vasiliadis, 2017 [54]	Children, adolescents and young adults	Not applicable	1–24	Canada	Manitoba, Ontario, Quebec, nova scotia	ICD-9 and ICD-10	Electronic health records	General practitioners, pediatricians, psychiatrists, or other specialists	1999–2012 (yearly brackets between 1999–2000 and 2011–2012)	Register
Wang, 2017 [14]	Preschool children, children and adolescents	145,018	0–18	Taiwan	Nationwide	ICD-9-CM	Electronic health records	Non explicit	2000–2011	Register
Young, 2015 [55]	Adults	3,4 million	18–	United States	Northern California	ICD-9	Electronic health records	Non explicit	January 1, 2010–December 31, 2010	Register
Bitsko, 2022 [56]	Preschool children, children and adolescents	114,476	3–17	United States	Nationwide	DSM-4?	Diagnosis of ADHD	Parents	2013–2019	Survey
Brault, 2012 [57]	Preschool children, children	13,904	3–9	Canada	Nationwide	DSM?	Diagnosis of ADHD	Person most knowledgeable about the child	2001–2001 (psychiatric diagnosis of ADHD)	Survey
Danielson, 2017 [58]	Children	17,889 (2007–2008); 19,897 (2011–2012)	2–5	United States	Nationwide	DSM-5	Diagnosis of ADHD	Parents	2007–2008, 2011–2012	Survey
Danielson, 2018 [59]	Children and adolescents	45,736	2–17	United States	Nationwide	DSM	Diagnosis of ADHD	Parents	2016	Survey
Hesson, 2018 [60]	Adults	16,957	20–64	Canada	Nationwide	DSM–4; ICD-10	Diagnosis of ADHD	Self–rate	2012	Survey
Parasurman, 2020 [61]	Children	21,539	6–11	United States	Nationwide	Non explicit	Diagnosis of ADHD	Parents	2016–2017	Survey
Shehadeh-Sheeny, 2023 [62]	Children	517	7–10	Israel	Northern Israel	DSM-5	Diagnosis of ADHD	Parents and homeroom teachers	October 2021–May 2022	Survey
Ten Have, 2023 [63]	Adults	6,194 (NEMESIS–3)	18–75	Netherlands	Nationwide	DSM-4, DSM-5	Diagnosis of ADHD	Individuals were interviewed (face–to–face diagnostic interview)	2007–2009–2019–2022 NEMESIS–3: November 2019 to March 2022	Survey
Visser, 2014 [64]	Children and adolescents	73,123 (2007) and 76,015 (2011)	4–17	United States	Nationwide	Non explicit	Diagnosis of ADHD	Responding parent or guardian	2003–2011	Survey
Yang, 2022 [65]	Children and adolescents	14,983	3–17	United States	Nationwide	DSM-5	Diagnosis of ADHD	Parents	2019–2020	Survey

ICD-10, The International Classification of Diseases 10th Revision; ICD-10-GM, German modification of the International Classification of Diseases 10th Revision; ICD-9, The International Classification of Diseases 9th Revision; ICD-9-CM, The International Classification of Diseases 9th Revision Clinical Modification; DSM-4, DSM-IV: Diagnostic and Statistical Manual of Mental Disorders, fourth dition; DSM-5: Diagnostic and Statistical Manual of Mental Disord ers, fifth edition; ICD-10-CA: International Classification of Diseases, 10th Revision, with Canadian Enhancements; F, females; M, males.

The included studies focused mostly on the population of preschool children, children in elementary school, adolescents, and young adults.

Diagnoses in these studies were determined through the analysis of electronic health records, population-based and administrative data, and/or health insurance data (register studies). They provided information through surveys (survey studies) and interviews with children, parents/guardians and/or teachers, using measures that included DSM and/or ICD diagnostic criteria (one-stage and two-stage clinical studies).

### Overall prevalence of ADHD

The prevalence estimates of ADHD reported in the included studies varied substantially and offered a range of heterogeneous data. Heterogeneity was attributed mostly to the studies’ methodological characteristics and age categories. Subgroup analysis showed that the prevalence estimates differed significantly across different age categories, except for the meta-analysis done for one-stage clinical studies. Furthermore, the remaining heterogeneity within subgroups remained high, above 90% in almost all age subgroups and all study types.

### Prevalence of ADHD in register studies and surveys

In total, 42 studies assessed the prevalence of ADHD by analyzing different databases or conducting a health survey. These studies are presented in Table 1.

The primary data source for the 32 studies was a review of electronic health and/or administrative records for a cohort of patients. Then, 13 studies were conducted in North America, 14 in Europe, 3 in Asia, and 2 in the Middle East.

The majority of the studies included children and/or adolescents. Twelve studies also included young adults and/or adults. In the study of Bachmann et al. [27], the frequency of ADHD diagnosis in the age range of 18–69 years was 0.2% (M: 0.3%; F: 0.2%) in 2009 and 0.4% (M: 0.5%; F: 0.3%) in 2014. In the study of Burd et al. [31], after 18 years of age, ADHD was diagnosed in 2% of the population. Hauck et al. [37] concluded that the overall prevalence of ADHD was 5.4%, with a higher prevalence in males but in not females, in older cohorts, although the reasons for the higher prevalence in older age categories were unclear. In the study of Chung et al. [33] 1.12% adult patients received diagnoses of ADHD [33]. Prevalence increased from 0.43% in 2007 to 0.96% in 2016. Among children aged 5–11 years, the prevalence increased from 2.96% in 2007 to 3.74% in 2016 [33]. Most recent studies focused on the increase in the prevalence of patients diagnosed with ADHD and on the other hand underdiagnosis and undertreatment of ADHD. Giacobini et al. [40] reported the annual prevalence of diagnosed ADHD in Sweden as 1.1 per 1,000 persons in 2006, which increased to 4.8 per 1,000 persons in 2011 (in all ages). They also concluded that the mean age of patients diagnosed with ADHD increased between 2006 and 2011, with a slight increase in the mean age of new diagnoses (21.2 in 2007 and 22.3 in 2011) [40]. In the cohort study by McKechnie et al. [47], the prevalence of ADHD diagnoses is highest among children. The overall proportion of ADHD diagnoses in male children aged 3–17 years was 175 per 10 000 (95% CI 174–177), or 1.8%; in female children aged 3–17 years, it was 37.7 per 10 000 (95% CI 37.1–38.3), or 0.4%. For male adults aged 18–99 years, the overall proportion was 28.8 per 10 000 (95% CI 28.6–29.0), or 0.3%; for female adults aged 18–99 years, it was 7.2 per 10 000 (95% CI 7.1–7.3), or 0.07% [47]. Proportionally, rates increased among adults from 2000 to 2018. However, the authors found evidence of increases in the proportion of people with ADHD diagnoses between 2000 and 2018 [47]. Ramos-Quiroga et al. [50] reported that ADHD continues to be underdiagnosed and undertreated, particularly in female adults. The prevalence of ADHD in children was estimated at 4.9%, whereas that in adults was estimated at 0.1%.

In the study of Holden et al. [42], diagnostic criteria were not explicitly defined; McKechnie et al. [47] used the Read code system. DSM diagnostic criteria were used in the study by Brook et al. [29] Fast et al. [38], Habdank-Kolaczkowski [41], and Davidovitch et al. [34]. In other studies, ICD-9 and/or ICD-10 criteria were used.

Eight survey studies were conducted in North America (Canada and the United States). Five studies assessed ADHD in children and adolescents (3–17 years old). In these studies, the person most knowledgeable about the child, parent, or guardian answered questions about the presence of a child’s psychiatric diagnosis of ADHD, confirmed by a doctor or other healthcare provider. Children and/or adolescents were subjects in eight studies. Ten Have et al. [63] and Hesson et al. [60] examined the prevalence of ADHD in adults. In both studies, ADHD prevalence was higher in men than in women (3.7% vs. 2.7% in women and 3.2% overall [63]; *n* = 287, 58.8% vs. *n* = 201, 41.2% in women [60]). Diagnostic criteria were not explicitly defined in two studies – Parasurman et al. [61] and Visser et al. [64]. In other studies, the DSM criteria (or in combination with ICD criteria) were used.

### Prevalence of ADHD in one-stage clinical studies

Twenty-five studies provided data on ADHD prevalence by one-stage clinical studies. These studies are presented in Table 2. Data collection tools used were: The Mini International Neuropsychiatric Interview (MINI), Development and Well-Being Assessment, Diagnostic Interview Schedule for Children (DISC), Diagnostic Interview for Children and Adolescents (DICA), INCLEN Diagnostic Tool for Attention-Deficit Hyperactive Disorder, DSM-IV questionnaire, The Kiddie Schedule for Affective Disorders and Schizophrenia (K-SADS), ADHD Rating Scale, Child Behavior Checklist, Teacher’s Report Form, Youth Self-Report, Peer-Relations Questionnaire, and Revised Behavior Problem Checklist.

**Table 2.** Descriptive data for studies included in the systematic review – one-stage clinical studies

First author and year of publication	Study population category	Sample size	Age range	Country	Region/city	Diagnostic standard	Diagnostic measure	Informant(s)	Timeframe
Aliye, 2023 [66]	Children and adolescents	504	6–17	Ethiopia	Jimma town	DSM-IV	VADHD+ structured questionnaire	Parents or caregivers, BSc Psychiatry, clinical nurses	August–September 2021 among
Anderson, 1987 [67]	Children	792	11	New Zealand	Dunedin	DSM-III	DISC-C	Parents, teachers, children; psychiatrists with experience in child psychiatry	February 1983–March 1984
Arora, 2018 [68]	Children and adolescents	3,977	6–9	India	Five regions in India	DSM-IV-TR	INDT-ADHD	Clinicians	December 5, 2011–September 27, 2012
Ashenafi, 2001 [69]	Children	1477	5–14	Ethiopia	Butajira district	None or non explicit	DICA-R	Caretakers, interviewers,	January–December 1998
Bøe, 2021 [70]	Children and adolescents	2,043	10–14	Norway	Bergen	DSM-IV	DAWBA	Parents, guardians, children and teachers, highly trained and experienced clinical rater	2006
Chen, 2019 [71]	Children and adolescents	4,816	7–14	Taiwan	Nationwide	DSM-5-	K-SADS-PL	Children, parents; non explicit	2015–2017
Cuffe, 2001 [72]	Adolescents, young adults	3,419	16–22	United States	Southeastern	DSM-III-R	K-SADS	Adolescent and one parent; psychiatry residents and doctoral-candidate psychologists	1991–1994
Ercan, 2015 [73]	Children and adolescents	417	6–14	Turkey	Central district of Izmir	DSM-IV (ADHD-RS-IV); DSM (K-SADS-PL)	K-SADS-PL, ADHD-RS-IV, CBCL, TRF	Teachers, parents, child and adolescent psychiatry resident	Non explicit
Ercan, 2022 [74]	Children	5,842	8–10	Turkey	Nationwide	DSM-III-R and DSM-IV criteria (K-SADS-PL)	K-SADS-PL, DISC-IV	Parents, teachers; the study team	Academic year 2014–2015?
Farbstein, 2014 [75]	Adolescents	957	14–17	Israel	Nationwide	DSM-IV and ICD-10	DAWBA	Adolescents and mothers, trained lay interviewers	1/2004–3/2005
Ford, 2003 [76]	Children and adolescents	10,438	5–15	United Kingdom	Nationwide	DSM-IV	DAWBA	Parents, teachers, child and adolescent psychiatrist, trainee child and adolescent psychiatrist	1999
Francés, 2023 [77]	Children	289	6	Spain	Menorca	DSM-5-TR	Different instruments+ direct observation, clinical interviews	Pediatricians, nurses, child psychiatrist	December 2020, January, February, and March 2021
Froehlich, 2007 [78]	Children and adolescents	3,082	8–15	United States	Nationwide	DSM-IV	DISC+prior diagnosis	Caregivers, non explicit	2001–2004
Gomes, 2019 [79]	Adults	3,781	22–22	Brazil	Pelotas	DSM-IV and ICD-10	MINI	Adults; trained psychologists	2015–2016
Kadesjö, 1998 [80]	Children	409	7–7	Sweden	Middle-sized town Karlstad	DSM-III-H	Parent interview, observation of the child, examination of the motor skills, teacher interview, and Conners screening tool	Teachers, parents, children; pediatrician	August 1992
La Maison, 2018 [81]	Children	3,562	11–11	Brazil	Pelotas, state of Rio Grande do Sul	DSM-IV, DSM-5 and ICD-10	DAWBA	Mothers or caregivers; trained psychologists	42,282
Leung, 2008 [82]	Adolescents	541	Grades 7, 8 and 9 students	China	Hong Kong	DSM-IV	DISC-IV, CBCL, YSR	Adolescents and their parents; non explicit	Non explicit
Maalouf, 2016 [83]	Adolescents	510	11–17	Lebanon	Beirut	DSM-IV and ICD-10	DAWBA, PRQ	Parents and adolescents, research team	3/2012–12/2012
McGee, 1990 [84]	Adolescents	962	15–15	New Zealand	Dunedin	DSM-III	DISC-C, RBPC	Adolescents, parents; research team	2/1987–5/1988
Moffitt, 2015 [85]	Adolescents and adults	1,037	11–38	New Zealand	Dunedin	DSM-5	DISC	Children, parent/teacher, adults, trained interviewers with mental-health-related tertiary qualifications and clinical experience	2010?
Petresco, 2014 [86]	Children	3,585	6–6	Brazil	Pelotas, southern Brazil	DSM-IV and ICD-10	DAWBA	Mothers or caregivers, psychologists	2010?
Pillai, 2008 [87]	Adolescents	2,048	12–16	India	Goa	ICD-10 and DSM-IV	DAWBA	Adolescents, parents, experienced child and adolescent psychiatrist	October 2002–May 2003
Safavi, 2019 [88]	Children and adolescents	1,038	6–18	Iran	Chaharmahal and Bakhtiari Province	DSM-IV	K-SADS-PL	Children and parents, clinical psychologists	2017
Shekim, 1985 [89]	Children	114	9–9	United States	Two rural Midwestern counties	DSM-III	DISC	Children and parents, mental health professionals: graduate students in psychology and school counselors with M.A. degrees	Non explicit
Talepasand, 2019 [90]	Children and adolescents	1,037	6–18	Iran	Semnan province	DSM-IV	K-SADS-PL	Children, adolescents, parents, clinical psychologists	2017

DSM-III, The Diagnostic and Statistical Manual of Mental Disorders, Third Edition; DSM-III-R, The Diagnostic and Statistical Manual of Mental Disorders, Revision, Third Edition; DSM-IV-TR, Diagnostic and Statistical Manual of Mental Disorders Text Revision Fourth Edition; DSM-IV, The Diagnostic and Statistical Manual of Mental Disorders, Fourth Edition; DSM-5, The Diagnostic and Statistical Manual of Mental Disorders, Fifth Edition ICD-9, The International Classification of Diseases 9th Revision; ICD-10, The International Classification of Diseases 10th Revision; DISC-C, Diagnostic Interview Schedule for Children, Child Version; INDT-ADHD, INCLEN Diagnostic Tool for Attention-Deficit Hyperactivity Disorder; DICA-R, Diagnostic Interview for Children and Adolescents; DSM-IV questionnaire, The Diagnostic and Statistical Manual of Mental Disorders, Fourth Edition questionnaire; DAWBA, Development and Well-Being Assessment; K-SADS-PL The Kiddie Schedule for Affective Disorders and Schizophrenia for School-Age Children-Present and Lifetime Version; K-SADS, The Kiddie Schedule for Affective Disorders and Schizophrenia; ADHD-RS-IV, Attention-Deficit/Hyperactivity Disorder Rating Scale IV, teacher and parent forms; CBCL, Child Behavior Checklist; TRF, Teacher’s Report Form; DISC-IV, Diagnostic Interview Schedule for Children, Version IV; DISC, Diagnostic Interview Schedule for Children; YSR, Youth Self-Report; PRQ, Peer-Relations Questionnaire; RBPC, Revised Behavior Problem Checklist.

Three studies were conducted in North America, three in South America, four in Europe, seven in Asia, two in Africa, three in Oceania, and three in the Middle East.

Gomes et al. [79] assessed the prevalence of ADHD in young adults, other studies evaluated ADHD prevalence in children and/or adolescents (Cuffe et al. [72] and Moffitt et al. [85] included also adults). Gomes et al. [79] used data from the 1993 Pelotas Birth Cohort (Brazil) and assessed the prevalence of psychiatric diseases at 22 years using the MINI. The ADHD prevalence was 4.5% (M: 4.1%, F: 4.8%) [79]. The weighted prevalence of DSM-III-R ADHD in the study of Cuffe et al. [72] was 1.51% (males: 2.62%, females: 0.54%), with significant association for gender (male). Moffitt et al. [85] reported 3% prevalence in the adult-ADHD group and gender balance.

Ashenafi et al. did not explicitly defined diagnostic criteria; however, DICA was used (structured interview for school-age children, based on the DSM criteria [69]). In other studies, the DSM criteria (or in combination with ICD criteria) were used.

### Prevalence of ADHD in two-stage clinical studies

Fifty studies provided data on ADHD prevalence through two-stage clinical studies. These studies are presented in Table 3. In the first stage, a questionnaire screening phase was conducted, followed by a diagnostic interview.

**Table 3.** Descriptive data for studies included in the systematic review - two-stage clinical studies

First author and year of publication	Study population category	Sample size	Age range	Country	Region/city	Diagnostic standard	Diagnostic measure	Informant(s)	Timeframe	Number of participants - stage 1	Number of participants - stage 2
Almqvist, 1999 [91]	Children	5,813	8–9	Finland	Nationwide	DSM-III-R	RA2, RB2, CDI+semistructured parent interview	Children, parents, teachers	1989 (first stage)	5813	435
Alyahri, 2008 [92]	Children	1,21	7–10	Yemen	City of Mukalla or the rural area of Tuban	DSM-IV	SDQ+DAWBA (2-phase design in an urban area and a 1-phase design in a rural area)	Parents, teachers	2002/2003 academic year	1210	465
Ambuabunos, 2011 [93]	Children	1,473	6–12	Nigeria	Egor Local Government Area of Edo State	DSM-IV	DBD + interviews	Children, parents, and teachers	February 2006–August 2006	1473	201
Amiri, 2010[94]	Children	1,658	7–15	Iran	Tabriz (North-West)	DSM-IV-TR	CBRS + clinical assessment	Adolescents, trained teachers, adolescent psychiatrist	2007-2008	1658	216
Amiri, 2014 [94]	Adults	400	18–45	Iran	Tabriz (North-West)	DSM-IV-TR	CAARS + clinical interview	Adults	2009	400	21
Bansal, 2011 [95]	Children and adolescents	982	10–15	India	City of North India	ICD-10	CPMS + clinical assessment	Respective principals and parents of the children, children	July–August 2010	982	199
Benjasuwantep, 2002 [96]	Children and adolescents	353	Primary school students	Thailand	Bangkok	DSM-IV	Raven’s progressive matrices, CPRS + clinical interview	Parents	Non-explicit	353	Non-explicit
Bianchini, 2013 [97]	Children and adolescents	6,183	5–15	Italy	Urban area of Syracuse	DSM-IV-TR	SDAI+K-SADS-PL, SNAP-IV, WISC-III, DDE, M.T. reading test	Teachers	9/2010–12/2011	6183	332
Bishry, 2018 [98]	Adolescents	925	12–15	Egypt	Eastern Cairo	DSM-IV	CASS:S+K-SADS-PL	Schoolteachers, physicians, participants, psychiatrist	Non-explicit	925 adolescents screened by the Conners-Wells’ Adolescent Self-Report Scale-Short form (CASS:S)	87
Bosch, 2021 [99]	Children and adolescents	6,834	5–17	Spain	Catalonia	DSM	CBCL, TRF, CPRS-R:S, YSR+K-SADS/PL, WISC	Parents, teachers, students	2011 (duration over 6 academic years)	6,834	2,298
Canals, 2018 [100]	Preschool children	1,104	3–6	Spain	Catalonia (rural areas of the Tarragona province and urban area near Barcelona)	DSM-IV	ECI-4+clinical interviews and observations	Parents and teachers, children	Non-explicit	1,104 (851)	516
Catherine, 2019 [101]	Children	3,253	8–11	India	Kancheepuram district	DSM-5	CBRS	Children, caregivers and teachers; investigators	Non-explicit	3,253	Non-explicit
Costello, 2003 [102]	Children and adolescents	6,675	9–16	United States	Western North Carolina	DSM-IV	CBCL + CAPA	Residents of the study area; parents, children	July-December 1998 (a random sample of the whole cohort)	6,675	1,420
Daniel, 2024 [103]	Children and adolescents	722	6–18	Mozambique	Nampula City	DSM-5	SNAP-IV + KSADS-PL	Child and adolescent psychiatrist; youths and parents	2019	722	132
Deivasigamani, 1990 [104]	Children	755	8–12	India	Madurai city; Tamil Nadu	ICD-9	Rutter B Scale + clinical evaluation and parental interview	Parents and teachers	Non-explicit	755	207
Dodangi, 2014 [105]	Children and adolescents	371	6–18	Iran	Paveh	DSM-IV TR	CSI-4, CBCL+K-SADS-PL	Parents, students	2012–2013	379	141
Donfrancesco, 2015 [106]	Children and adolescents	1,887	9.58 ± 1.84 (ADHD, diagnosed by psychiatric interview based on K-SADS-PL)	Italy	Tuscany and Latium	DSM-IV	SDAI+SDAG, K-SADS-PL	Teachers and parents, children, experienced child psychiatrist	School year 2002–2003	1,887	27
Elberling, 2016 [107]	Children	5,898	5–7	Denmark	Area around the city of Copenhagen	ICD-10	SDQ + DAWBA	Parents and preschool teachers, clinicians	Non-explicit	5,898	1,585
Ercan, 2013 [108]	Children	1,455	8–12	Turkey	Izmir	DSM-IV	T-DSM-IV-S+K-SADS-PL,WISC-R, interview	Parents and teachers, trained psychologist, nurse	4 years (4 waves) 2008–2011	1,455	86 positive screening, 85 negative screening
Farahat, 2014 [109]	Children	1,362	6–12	Egypt	Menoufia governorate (Menouf city, Monshaat Sultan village, Menouf district)	DSM-IV	WISC, CBRS + clinical interview	Parents and teachers; family medicine consultants, psychologist, psychiatric consultant	Non-explicit	1,362	Non-explicit
Fayyad, 2017 [23]	Adults	11,422	18–44	In ten countries in the Americas, Europe, and the Middle East	Belgium, Colombia, France, Germany, Italy, Lebanon, Mexico, The Netherlands, Spain and the United States	DSM-IV	DISC + ACDS (United States only), clinical reappraisal interviews (United States only)	Trained lay interviewers, four experienced clinical interviewers (all PhD-qualified clinical psychologists)	2001–2003	11422	154 (clinical)
Gau, 2005 [110]	Adolescents	1,070	13–15	Taiwan	South Taiwan	DSM-IV	K-SADS+K-SADS-E, CBCL	Nurses, psychologists, social workers, and psychiatric residents; consultant child psychiatrists	1994–1995, 1995–1996 1996–1997	1,070	Follow-up 1,051 and 1,035
Hebrani, 2007 [111]	Preschool children	1,083	5–6	Iran	Mashhad, north-East of Iran	DSM-IV	CBRS+K-SADS-PL	Teachers and parents, children; psychiatrist/highly trained interviewer	Non-explicit	1,083	Non-explicit
Heiervang, 2007 [112]	Children	9,430	8–10	Norway	Bergen	DSM-IV, ICD-10	SDQ + DAWBA	Parents and teachers; child Psychiatrists	2002–2003	9,430	1,011
Huang, 2017 [113]	Children	2,959	7–12	China	Shantou, Districts of Longhu and Jinping	DSM-5	CBRS + clinical interview	Students, teachers and parents; psychiatrists	9/2013–4/2014	2,959	651
Jin, 2014 [114]	Children and adolescents	5,648	5–15	China	Zhabei District, Shanghai	DSM-IV	DSM-IV ADHD questionnaire + clinical interview	Students, parents; child psychiatrists	random cluster sampling 4-5/2009, clinical interviews 12/2009–1/2010	5,648	Non-explicit
Kessler, 2006 [113]	Adults	3,199	18–44	United States	Nationwide	DSM-IV	DIS+ACDS	Adults, clinical psychologists	Non-explicit	3,199	154
Kusi-Mensah, 2019 [115]	Children and adolescents	71,929	303	Ghana	Asawasi, a sub-district of Kumasi	DSM	CBCL, TRF+K-SADS-PL	Parents, class teachers, psychiatrist, resident doctor in psychiatry	2016	303	173
Li, 2022 [116]	Children and adolescents	71,929	6–16	China	Nationwide	DSM-IV	CBCL + MINI	Primary caregivers	10/2014–3/2015	71,929	17,524
Lynch, 2006 [117]	Adolescents	723	12–15	Ireland	Dublin	DSM-IV	SDI, SDQ+K-SADS-PL	Young person, parent	Non-explicit	723	314 (140 individuals identified as being “at risk” [67 males, 73 females], and a comparison group [N = 174])
Matandika, 2022 [118]	Children, adolescents	354	6–17	Malawi	Blantyre City	DSM-5	SDQ+ K-SADS-PL	Medical doctor	Children, adolescents; parents or guardians, professional nurses	354	Non-explicit
Matte, 2015 [119]	Adults	4,000	18–19	Brazil	Pelotas	DSM-5, ICD-10	ASRS + structured interview	Adults; trained psychologists	2011-2012	4,000	1,329
Michielsen, 2012 [120]	Adults	1,494	55–85	Netherlands	Nationwide	DSM-IV-TR	Screening questionnaire developed by Barkley et al. + DIVA	Lay interviewers	1992–1993, 1995–1996, 1998–1999, 2001–2002, 2002–2003, 2005–2006	1,494	231
Mohammadi, 2016 [121]	Children and adolescents	9,636	6–18	Iran	Five provinces of Iran: Tehran, Shiraz, Isfahan, Tabriz and Mashhad	DSM-IV	SDQ+K-SADS-PL	Parents, children; clinical psychologists	Non-explicit	9,636	2,051
Montiel, 2008 [122]	Children and adolescents	1,535	4–12	Venezuela	Maracaibo county	DSM-IV	CPRS-R, CTRS-R + clinical assessment	Parents, teachers. Psychologists	Non-explicit	1,535	233
Mulu, 2022 [123]	Children and adolescents	365	6–17	Ethiopia	Shewa Robit town	DSM-IV	Structured and pretested questionnaires + MINI kid	Psychiatry nurses, guardians/parents, psychiatry Professional	Feb 1-March 30, 2020	365	
Nazeer, 2022 [124]	Children	1,125	6–10	Sri Lanka	Colombo District	DSM-5	SNAP-IV P/T-S, SAQ, IAQ+	Teachers, Consultant Child and Adolescent Psychiatrist	July 2019–February 2020	1,125	1,125
Nomura, 2014 [125]	Preschool children	583	5	Japan	Kanie-cho, in Japan’s Aichi Prefecture	DSM-IV-TR	Age 5 exam + clinical evaluations during follow-ups (post-examination consultations, preschool visits, and group rehabilitation)	Child’s guardian, kindergarten teacher, child psychiatrist, clinical psychologists, public health nurse	4/2009–3/2011	583	83
Patil, 2013 [126]	Children and adolescent	257	5–14	India	Mumbai	DSM-IV	Diagnostic interview + clinical interview	Interviewer, psychiatrist	Non-explicit	257	
Pineda, 2003 [127]	Children and adolescents	330	4–17	Colombia	Manizales, Antioquia	DSM-IV (ADHD checklist based on DSM-IV criteria)	ADHD symptoms checklist, CBRS, BASC + clinical examination	Parents, teachers, postgraduate students of psychology and education, under the supervision of a neuropsychologist, certified neurologist and a certified psychiatrist	Non-explicit	330	Non-explicit
Puura, 1998 [128]	Children	3,206	8–9	Finland	Helsinki, Tampere, and Turku	DSM-III-R	The Rutter scale A2 for parents, the Rutter scale B2 for teachers, CDI+IWI, DISC-C	Children, parents, teachers; child psychiatrists	1989/1990	3206	279
Rohde, 1999 [129]	Adolescents	1,013	12–14	Brazil	Porto Alegre	DSM-IV	screening instrument + clinical interview	Adolescents, parents, trained research assistants, experienced child and adolescent psychiatrist	Non-explicit	1013	191
Sans, 2021 [130]	Preschool children and children	3,727	3-5; 10–12	Spain	Province of Tarragona	DSM-5	CBRS+K-SADS-PL, WISC-IV, Trail Making Test	Parents and teachers	2014–2019	3,727	781
Shen, 2018 [131]	Children and adolescents	17,071	6–16	China	Two cities in the central part of Hunan province (South China)	DSM-IV	CBCL+MINI	Students, parents, teachers (alternative informants), psychiatrists	Non-explicit (2015?)	17,071	3465 participants with positive scores+10% participants with negative scores
Smalley, 2007 [132]	Adolescents	6,622	16–18	Finland	Northern Finland	DSM-IV	SWAN + clinical assessment	Adolescents and their parents; mental health specialists trained	Non-explicit	6,622	464
Suvarna, 2009 [133]	Preschool children	1250	4–6	India	Mumbai	DSM-IV	CBRS + clinical interview	Teachers and parents	Non-explicit	1250	
Umar, 2018 [134]	Adolescents	487	11–19	Nigeria	Jos	DSM-IV	K-SAD-PL + Raven’s SPM, CGAS, K-SADS-PL	Students, (parents), lead Author and resident doctors in psychiatry	Non-explicit	487	Non-explicit
Wagner, 2017 [135]	Adolescents	3,477	10–18	Austria	Nationwide	DSM‑5	YSR, SCOFF + CDI-MD	Teachers, adolescents, psychologists, Medical doctor	Non-explicit	3,477	292
Xiaoli, 2014 [136]	Children and adolescents	8,848	6–17	China	Northeast China (Shenyang, Panjin, and Benxi)	DSM-IV	SDQ+DAWBA	Children, mothers and teachers, trained layman interviewers, child psychiatrists	Non explicit	8,848	1,824 (student) 1,535 (parent)
Zorlu, 2020 [137]	Children and adolescents	1,439	6–14	Turkey	Denizli	DSM-IV	T-DSM-IV-S+K-SADS-PL	Children, parents and teachers	2/2011–5/2011	1,439	141

DSM, The Diagnostic and Statistical Manual of Mental Disorders; DSM-III-R, The Diagnostic and Statistical Manual of Mental Disorders, Revision, Third Edition; DSM-IV, The Diagnostic and Statistical Manual of Mental Disorders, Fourth Edition; DSM-5, The Diagnostic and Statistical Manual of Mental Disorders, Fifth Edition; DSM-IV-TR, Diagnostic and Statistical Manual of Mental Disorders Text Revision Fourth Edition; ICD-9, The International Classification of Diseases 9th Revision; ICD-10, The International Classification of Diseases 10th Revision; CAARS, The Conners’ Adult ADHD Rating Scale; CBRS, The Conner’s index questionnaire; YSR, Youth Self-Report; SCOFF, a five-question screening tool designed to clarify suspicion that an eating disorder might exist rather than to make a diagnosis; CDI-MD, Childrens’ Diagnostic Interview for Mental Disorders; ASRS, The World Health Organization Adult ADHD Self-Report Scale; CBCL, The Achenbach’s Child Behavior Checklist; MINI, Mini International Neuropsychiatric Interview; SDQ, Strengths and Difficulties Questionnaire; DAWBA, Development and Well-Being Assessment; BASC, Behavior Assessment System for Children Colombian version; CBCL, Child Behavior Checklist; K-SADS-PL, Schedule for Affective Disorders and Schizophrenia for School-Aged Children; Present and Lifetime version; C-GAS, The Children’s Global Assessment Scale; WISCIII, The Wechsler Intelligence Scale for Children; ASSQ, The Autism Spectrum Disorder Screening Questionnaire; PDD, a checklist containing the diagnostic criteria for Pervasive Developmental Disorders; CASS:S, Conners-Wells’ Adolescent Self-Report Scale-Short form; RA2, the Rutter Parent Questionnaire; RB2, The Rutter Teacher Questionnaire; CDI, The Children’s Depression Inventory; CDI, The Children’s Depression Inventory; IWI, The semi-structured Isle of Wight Interview; DISC-C, The Diagnostic Interview Schedule for Children, child version; SWAN, The Strengths and Weaknesses of ADHD-Symptoms and Normal Behavior; TRF, Teacher’s Report Form; ACDS, Adult ADHD Clinical Diagnostic Scale, version 1.2; CPMS, Childhood Psychopathology Measurement Schedule; CSI-4, Children’s Symptom Inventory (CSI-4)-Parent Form; SDAG, The parent version of the ADHD rating scale (Scala per i Disturbi di Attenzione/Iperattività per Insegnanti or SDAG); DIVA, Diagnostic Interview for ADHD in Adults; DBD, Disruptive Behavior Disorder Rating Scale; Raven’s SPM, Raven’s progressive matrices test for estimation of intellectual functioning and observation for their behavior in the classrooms by one researcher; CGAS, Children’s Global Assessment Score, SDI, The Children’s Depression Inventory; SDAI, Rating Scale (ADHD Rating Scale for Teachers [Scala per i Disturbi di Attenzione/Iperattività per Insegnanti]; SNAP-IV, The Swanson, Nolan and Pelham Scale-Version IV; DDE, Battery for Evaluating Dyslexia and Dysorthography; M.T. reading test, a text, varying for each grade and phase of the scholastic year (initial, middle and final), that the participants are asked to read; K-CBCL, Korean Child Behavior Checklist; CPRS-R:S, The Conners’ parent rating scale - Revised, the Spanish version; CPRS, The Conners’ parent rating scale; CPRS-R, The Conners’ parent rating scale - Revised; ECI-4, The Early Childhood Inventory-4; K-SADS Schedule for Affective Disorders and Schizophrenia for School-Aged Children (Epidemiological version); T-DSM-IV-S, The Turgay DSM-IV Disruptive Behavior Disorders Rating Scale; WISC-R, The Wechsler Intelligence Scale for Children-Revised; CAPA, The Child and Adolescent Psychiatric Assessment; DIS, Diagnostic Interview Schedule; ACDS, The Adult ADHD; NTRS, The NIEHS Teacher Rating Scale; DISC, Diagnostic Interview Schedule for Children; CTRS-R, the Conners teacher rating scale - Revised; MINI Kid, Mini International Neuropsychiatric Interview for Children and Adolescents; SNAP-IVP/T-S, The validated and pretested Sinhala version of SNAP-IV Parent and Teacher rating scale; SAQ, The self-administered questionnaire; IAQ, interviewer administered questionnaire.

Two studies were conducted in North America, 4 in South America, 13 in Europe, 16 in Asia, 8 in Africa, and 6 in the Middle East. The prevalence of ADHD in children and/or adolescents was assessed in a great proportion of the studies included in this review. In five studies, the prevalence of ADHD in adults was assessed. In the study of Michielsen et al. [120], the estimated prevalence rate of syndromic ADHD in older adults was 2.8%; for symptomatic ADHD, the rate was 4.2%. Younger elderly adults (60–70 years) reported significantly more ADHD symptoms than older elderly adults (71–94 years) [120]. Fayyad et al. [23] assessed ADHD prevalence in adult respondents aged 18–44 years in 10 countries in the Americas, Europe, and the Middle East. Estimates of ADHD prevalence averaged 3.4% (range 1.2–7.3%), with a lower prevalence in lower-income countries (1.9%) compared with higher-income countries (4.2%) [23]. However, masked clinical reappraisal interviews administered to 154 US respondents in this study yielded a prevalence estimate of 5.2% for adult ADHD [23]. In the study of Matte et al. [119], ADHD prevalence in 18- to 19-year-old young adults was assessed. The prevalence of DSM-5 ADHD was 3.55% [95% confidence interval (CI) 2.98–4.12], and the estimated prevalence of DSM-IV ADHD was 2.8% [119]. Urban inhabitants of Tabriz (Iran) aged 18–45 years were selected for the study of Amiri et al. [94], and the prevalence of adult ADHD was estimated to be 3.8%. Men, when compared with women, were more likely to have ADHD (5.5% in men versus 2% in women) [94]. Kessler et al. [113] evaluated adult ADHD in a probability subsample of 18- to 44-year-old respondents in the National Comorbidity Survey Replication. The estimated prevalence of current adult ADHD was 4.4% [113]. The Diagnostic criteria used in two-stage clinical studies were preferably DSM criteria; ADHD prevalence ranged between 0.6% in the study by Puura et al. [128] and 16.4% in the study by Pineda et al. [127]. Heiervang et al. [112] used both the DSM and ICD criteria. Bansal et al. [95], Elberling et al. [107], and Deivasigamani et al. [104] used the ICD criteria, and the prevalence rates of kinetic syndrome/disorder were 6%, 1%, and 1.7%.

In two-stage clinical studies, the DSM diagnostic criteria were preferably used. Only in the studies of Bansal et al. [95], Elberling et al. [107], and Deivasigamani et al. [104], the ICD diagnostic criteria were used.

## Meta-analysis

**Figure 2.**
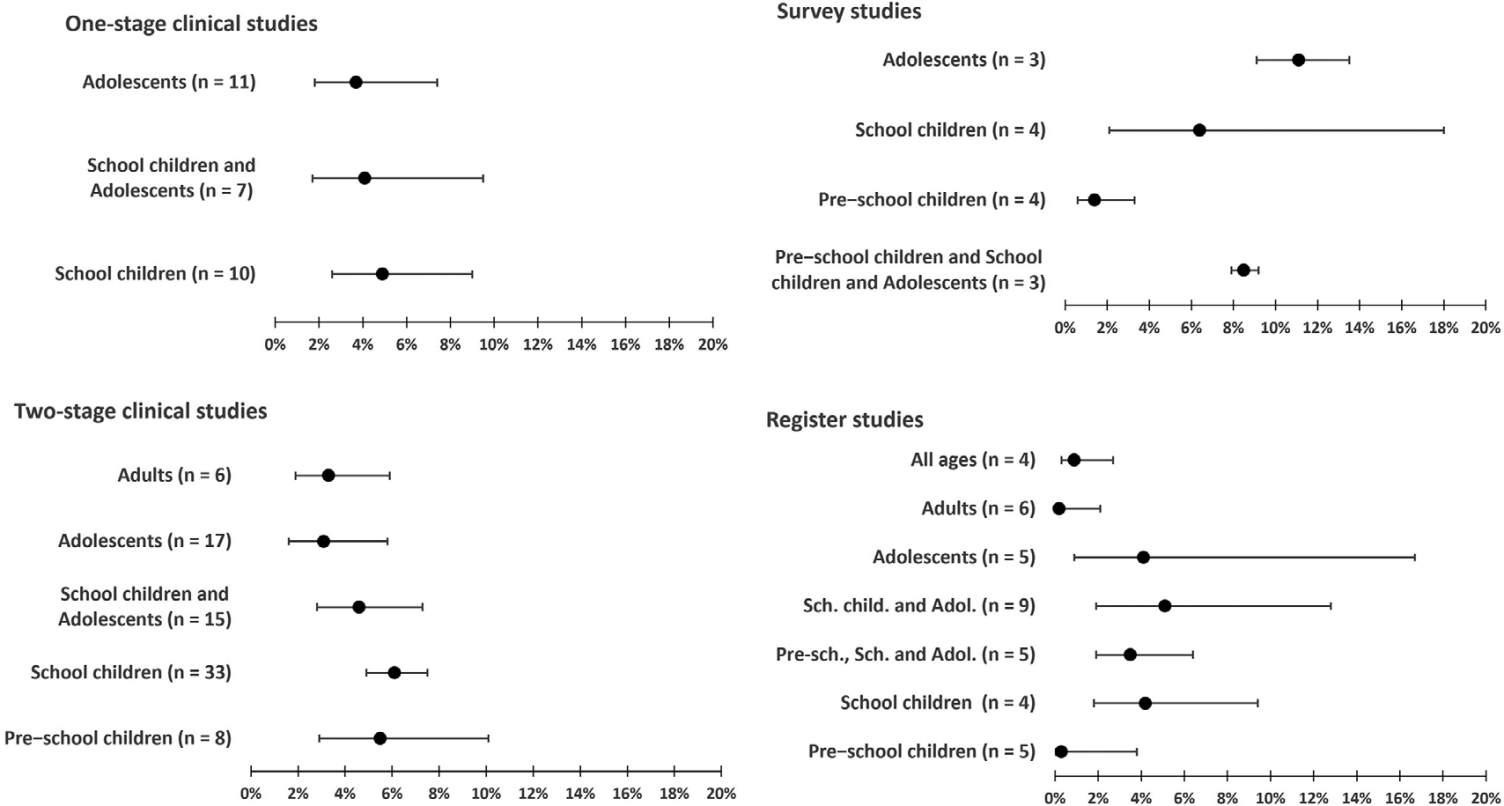
Summary of the meta-analysis results. Points with error bars represent the overall prevalence with 95% confidence intervals of each age subgroup (i.e., the diamond in a forest plot). Full forest plots are presented in the Supplementary material. Numbers in brackets represent the number of independent datapoints included in each age category.

In the meta-analysis, we analyzed separately each type of studies (register and survey studies, one-stage and two-stage clinical studies). Age categories were used as subgroups. Results are presented in Figure 2.

Studies were grouped in according to age. In the case of less than three studies in the subgroup, these studies were excluded. We identified 103 eligible studies for meta-analysis.

### One-stage clinical studies

We analyzed 28 independent datapoints (in total 22 studies). The overall prevalence of ADHD in one-stage clinical studies was 4.2%, 95% CI [2.9; 6.0]. In age subgroups, the results were 4.9%, 95% CI [2.6; 9.0] for school children; 3.7%, 95% CI [1.8; 7.4] for adolescents, and 4.1%, 95% CI [1.7; 9.5] for school children and adolescents.

### Two-stage clinical studies

We analyzed 79 independent datapoints (in total 49 studies). The overall prevalence of ADHD in two-stage clinical studies was 4.8%, 95% CI [4.0; 5.8]. In age subgroups, the results were 5.5%, 95% CI [2.9; 10.1] for preschool children; 6.1%, 95% CI [4.9; 7.5] for school children, 4.6%, 95% CI [2.8; 7.3] for school children and adolescents, 3.1%, 95% CI [1.6; 5.8] for adolescents, and 3.3%, 95% CI [1.9; 5.9] for adults.

### Survey studies

We analyzed 14 independent datapoints (in total 8 studies). The overall prevalence of ADHD in survey studies was 5.0%, 95% CI [2.9; 8.6]. In age subgroups, the results were 1.4%, 95% CI [0.6; 3.3] for preschool children; 8.5%, 95% CI [7.9; 9.2] for preschool and school children and adolescents; 6.4%, 95% CI [2.1; 18.0] for school children and 11.1%, 95% CI [9.1; 13.5] for adolescents.

### Register studies

We analyzed 38 independent datapoints (in total 24 studies). The overall prevalence of ADHD in register studies was 1.6%, 95% CI [0.9; 3.0]. In age subgroups, the results were 0.3%, 95% CI [0.0; 3.8] for preschool children; 4.2%, 95% CI [1.8; 9.4] for school children, 3.5%, 95% CI [1.9; 6.4] for preschool and school children and adolescents; 5.1%, 95% CI [1.9; 12.8] for school children and adolescents;4.1%, 95% CI [0.9; 16.7] for adolescents; 0.2%, 95% CI [0.0; 2.1] for adults; and 0.9%, 95% CI [0.3; 2.7] for all ages.

## Discussion

We conducted a comprehensive systematic review of studies evaluating worldwide prevalence rates of ADHD to understand better and explain the variability in ADHD prevalence data. The prevalence of ADHD was investigated across different age categories (children, adolescents, and adults). We compared the results among the different types of studies (register and survey studies, one-stage and two-stage clinical studies). These data must be interpreted cautiously due to the high variability in the analyses that are conducted. The results of this systematic review show that the overall prevalence of ADHD in register studies was 1.6%, 95% CI [0.9; 3.0], in survey studies was 5.0%, 95% CI [2.9; 8.6], in one-stage clinical studies was 4.2%, 95% CI [2.9; 6.0], and in two-stage clinical studies was 4.8%, 95% CI [4.0; 5.8]. The register studies exhibit the lowest estimate for ADHD prevalence, mostly because of inclusion of the entire population (e.g., children under 2 years old), while the other three types of studies show similar estimates. However, our results also indicate that the type of study methodology significantly impacts the estimation of ADHD prevalence. Register-based studies depend on the quality of different data collections (input data). Since ADHD still lacks biological markers, diagnosis relies on physicians’ education and practice [34], and cultural factors may also play a significant role in the identification of ADHD [11]. Therefore, ADHD prevalence can vary significantly. One-stage and two-stage clinical studies provide more comparable ADHD prevalence data.

Most of the studies included in our review utilized the DSM and/or ICD diagnostic criteria to diagnose ADHD. Additionally, different data collection tools, such as the DSM/ICD questionnaire, K-SADS, MINI, DAWBA, and DISC, were used in the included one-stage and two-stage clinical studies. When the diagnosis was based on DSM criteria, the edition of criteria affected the median prevalence in one-stage and two-stage clinical studies (lower average of prevalence medians in DSM-III vs. DSM-IV or DSM-5 criteria). However, the number of studies using the DSM-III criteria was significantly lower than those using updated diagnostic criteria.

Additional variations in the prevalence estimates can be attributed to the study populations. The prevalence of ADHD varies with age [9]. Previous systematic reviews have shown lower ADHD prevalence in adults compared to school children and adolescents [3, 12, 17–22]. Most of the studies in our review have been performed on children and adolescents. It is important to note that psychiatric diseases often persist into adulthood, leading to individual and collective social and economic burdens [1]. The differences in prevalence estimates between children and adults can be attributed to delayed brain maturation [139]. This delayed maturation, particularly in the frontal areas, may lead to a protracted developmental trajectory in ADHD, with brain function normalizing in young adulthood but declining prematurely in later years. Consequently, the neurobiological changes observed in ADHD during adulthood may contribute to the varying prevalence rates seen across different age groups [139].

Our study must be considered in the context of its limitations. First, several methodological issues need to be addressed. We limited our search to articles published in English for this review, while articles in other languages were not considered. Although most studies are published in English, we may have missed some articles (mainly in Chinese). Additionally, our search was limited to PubMed®, which may result in fewer articles being included. To address these limitations, replicating our systematic review, including other databases, such as Embase, is recommended. We also included only studies that included the general population, which might underrepresent local situations and represent the limitation of the evidence. Excluding other populations (e.g., institutionalized populations, “special needs” children) can lead to an underestimation of the actual burden of ADHD, which is necessary data for disease burden calculations. Second, we analyzed studies conducted at different time points and locations; this issue could be examined in a proper meta-regression analysis. Although we compared studies by type, not all methods used were standardized. Third, the number of studies was not evenly distributed in all geographical locations, especially in Africa, Oceania, and the Middle East. Additional studies in these regions are needed to confirm our results, calling for a meta-regression analysis in this area.

Despite some limitations, the results of our study have important implications. First, we included studies with clinically confirmed diagnoses of ADHD, which may contribute to the more objective and precise ADHD prevalence data compared to the systematic reviews that also included studies where ADHD diagnosis was based on nonclinical questionnaire results. Second, the type of study (methodology) significantly affects the estimation of ADHD prevalence. It is necessary to standardize diagnostic procedures to monitor the rates of ADHD efficiently. Third, our results can assist policymakers in better understanding ADHD, which is essential for resource planning and implementing global strategies for ADHD management worldwide. Our results can also encourage researchers to conduct additional analyses and systematic reviews, incorporating more databases.

In conclusion, the results from this systematic review and meta-analysis suggest that the type of study significantly affects the prevalence of ADHD. Standard diagnostic procedures to estimate ADHD prevalence may contribute to accurate prevalence data and provide high-quality, relevant, accessible, and up-to-date information for policymakers. Based on these findings, we propose that the current estimate of prevalence of ADHD should be based on the average of all study types.

## Supporting information

Popit et al. supplementary material Popit et al. supplementary material

## Data Availability

For supplementary material accompanying this article, visit cambridge.org/EPA.

## Supplementary material

The supplementary material for this article can be found at http://doi.org/10.1192/j.eurpsy.2024.1786.
