# Supplementary material for: Prevalence of attention-deficit hyperactivity disorder (ADHD): systematic review and meta-analysis
Source: Eur Psychiatry. 2024 Oct 9;67(1):e68. doi: 10.1192/j.eurpsy.2024.1786 (PMC11536208; doi:10.1192/j.eurpsy.2024.1786)

**Pubmed Search profile**

**1900/01/01:2024/2/01[Date - Create] AND (("Attention Deficit Disorder with Hyperactivity"[MeSH Terms] OR "Attention Deficit Disorder with Hyperactivity"[Title/Abstract] OR "Attention Deficit Disorders with Hyperactivity"[Title/Abstract] OR "attention deficit hyperactivity disorder*"[Title/Abstract] OR "attention deficit disorder*"[Title/Abstract] OR "ADHD"[Title/Abstract] OR "ADDH"[Title/Abstract] OR "minimal brain dysfunction*"[Title/Abstract] OR "minimal cerebral dysfunction*"[Title/Abstract] OR "hyperkinetic syndrome*"[Title/Abstract] OR "Hyperkinetic Disorder"[Title/Abstract] OR "hyperactive impulsiv*"[Title/Abstract] OR "hyperkines*"[Title/Abstract]) AND ("Epidemiology"[MeSH Terms] OR "Prevalence"[MeSH Terms] OR "epidemio*"[Title/Abstract] OR "prevalence*"[Title/Abstract] OR "Rate"[Title/Abstract] OR "Point Estimate"[Title/Abstract]))**

1900/01/01:2024/2/01[Date - Create] AND (("Attention Deficit Disorder with Hyperactivity"[MeSH Terms] OR "Attention Deficit Disorder with Hyperactivity"[Title/Abstract] OR "Attention Deficit Disorders with Hyperactivity"[Title/Abstract] OR "attention deficit hyperactivity disorder*"[Title/Abstract] OR "attention deficit disorder*"[Title/Abstract] OR "ADHD"[Title/Abstract] OR "ADDH"[Title/Abstract] OR "minimal brain dysfunction*"[Title/Abstract] OR "minimal cerebral dysfunction*"[Title/Abstract] OR "hyperkinetic syndrome*"[Title/Abstract] OR "Hyperkinetic Disorder"[Title/Abstract] OR "hyperactive impulsiv*"[Title/Abstract] OR "hyperkines*"[Title/Abstract]) AND ("Epidemiology"[MeSH Terms] OR "Prevalence"[MeSH Terms] OR "epidemio*"[Title/Abstract] OR "prevalence*"[Title/Abstract] OR "Rate"[Title/Abstract] OR "Point Estimate"[Title/Abstract]))

**Forest plot for One-stage clinical studies**


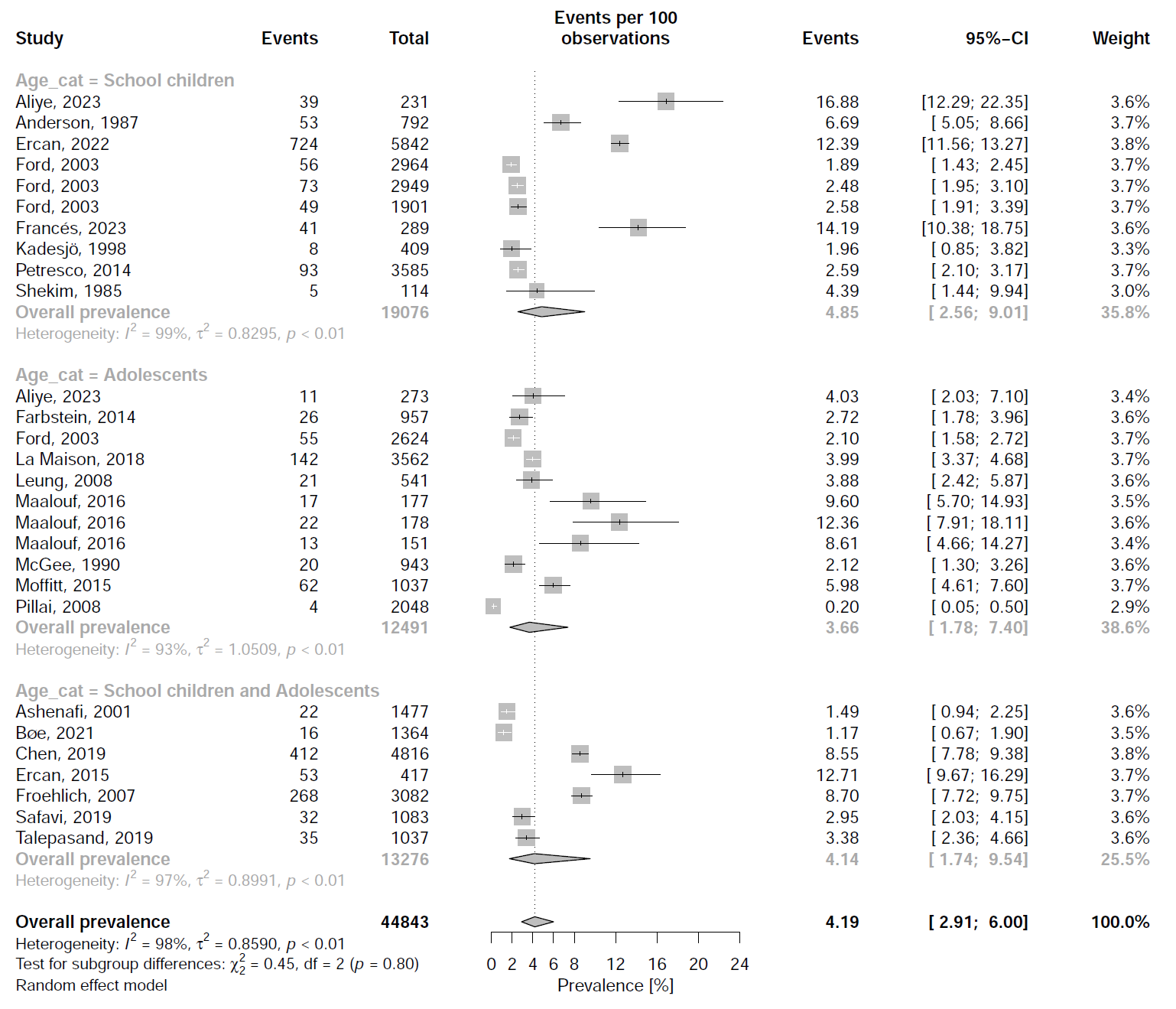


**Forest plot for Two-stage clinical studies (Part 1)**


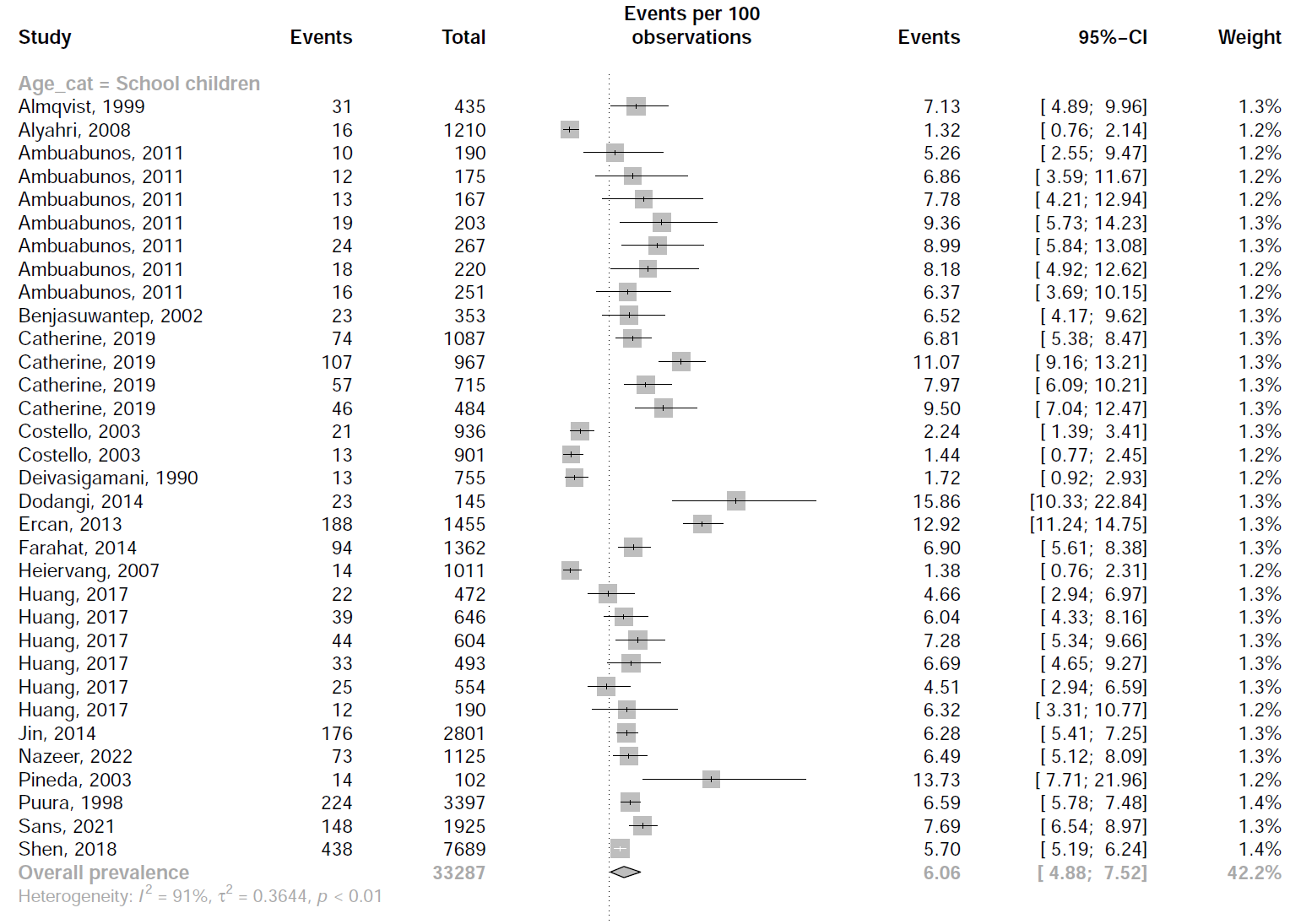


**Forest plot for Two-stage clinical studies (Part 2)**


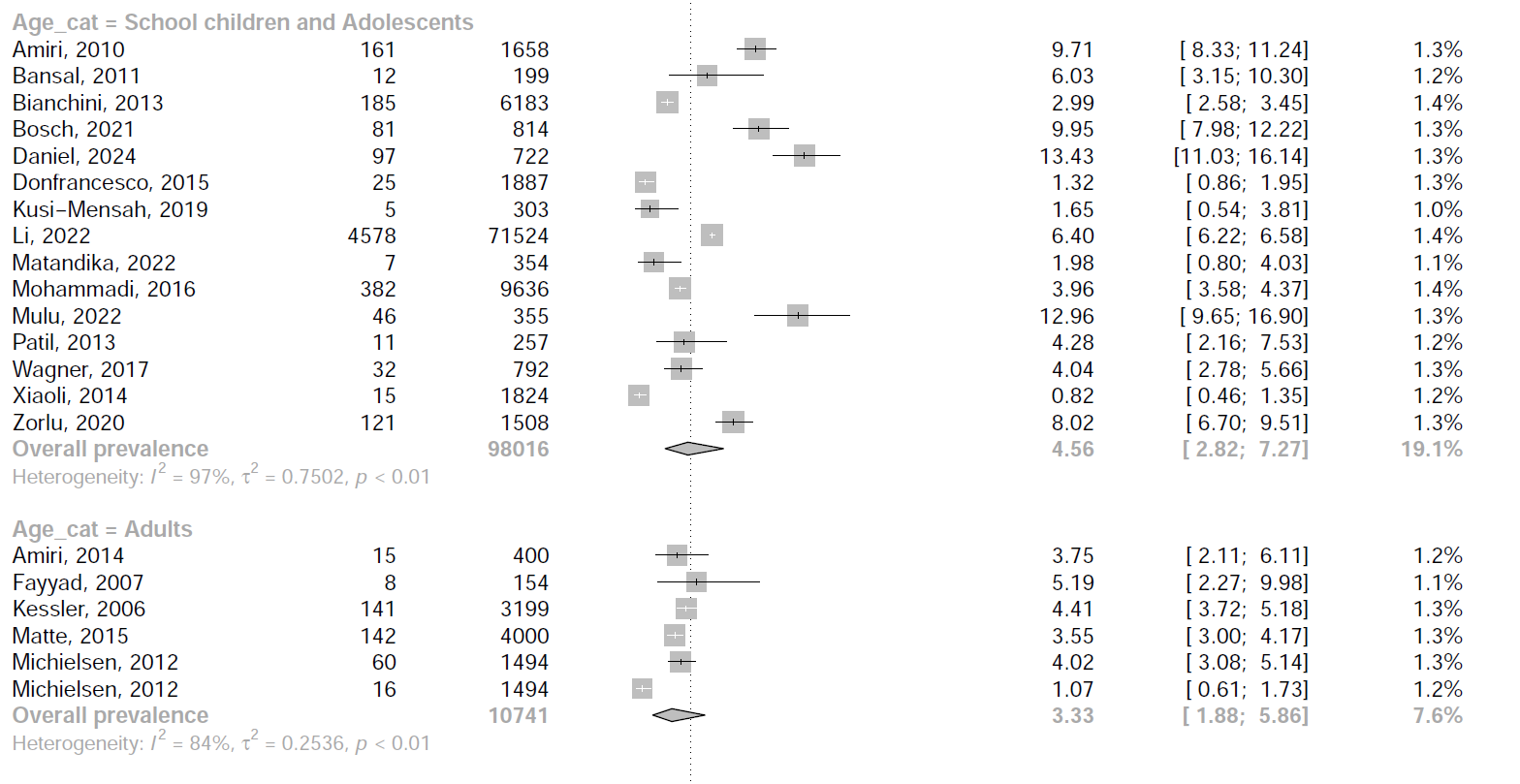


**Forest plot for Two-stage clinical studies (Part 3)**


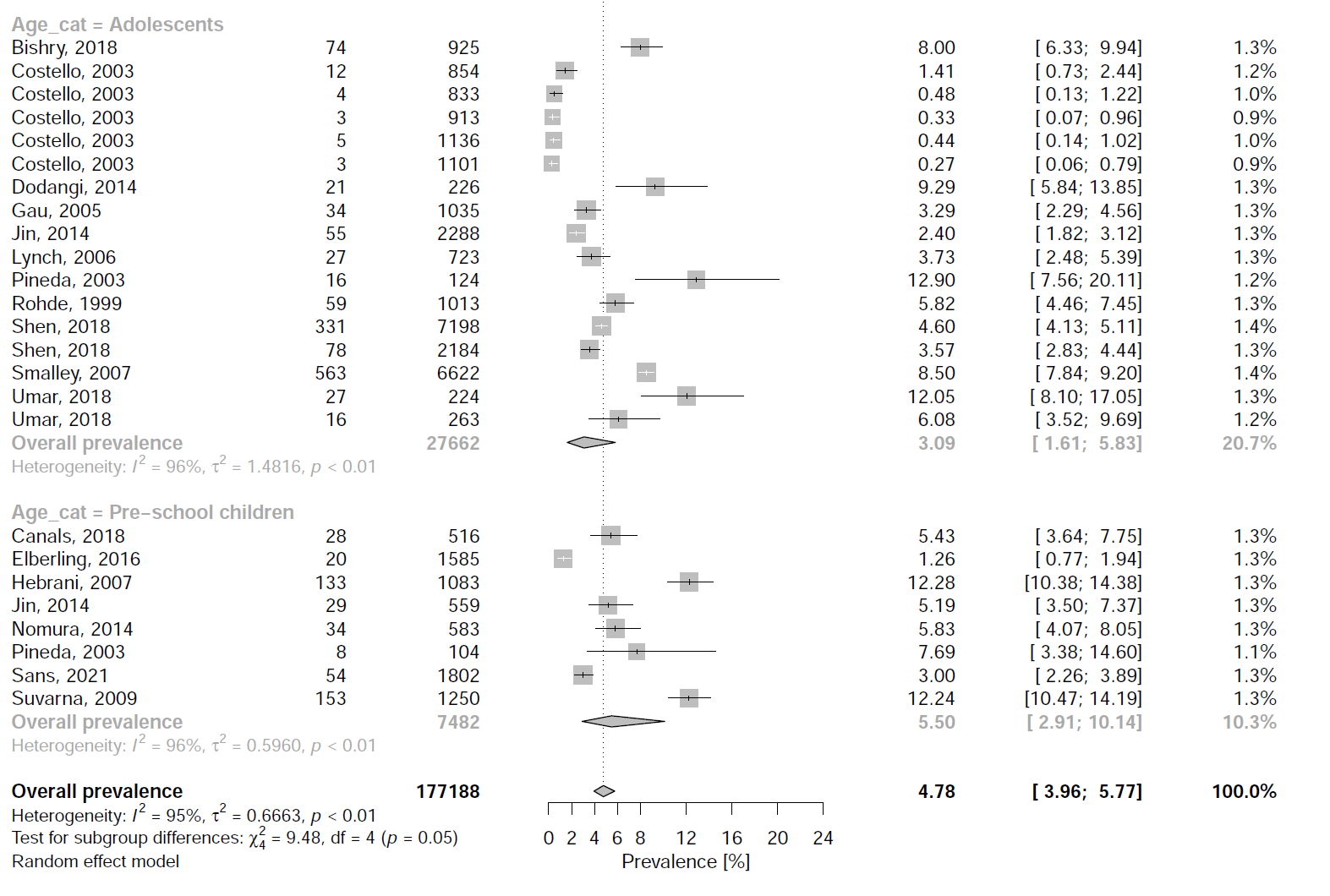


**Forest plot for Survey studies**


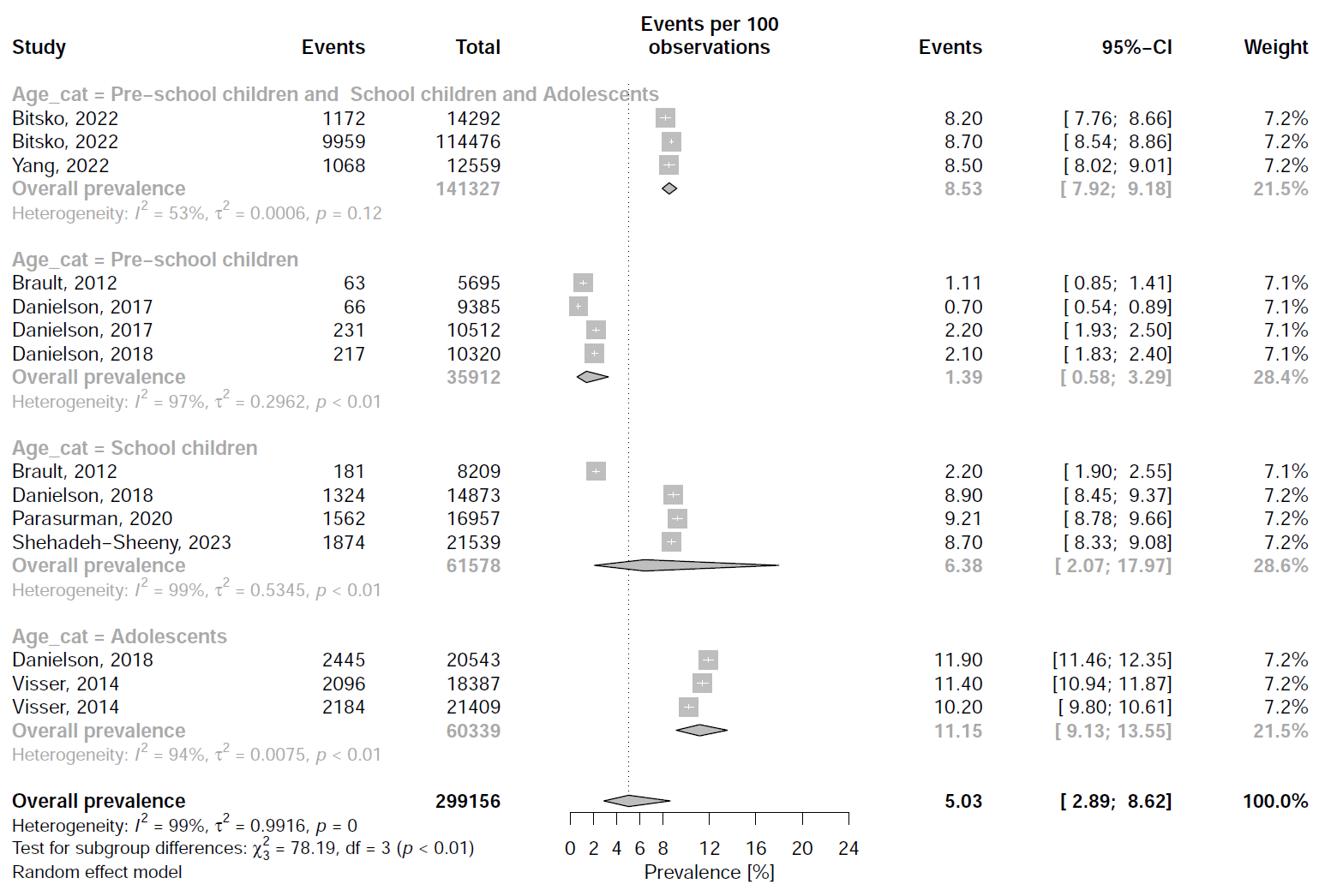


**Forest plot for Registry studies (Part 1)**


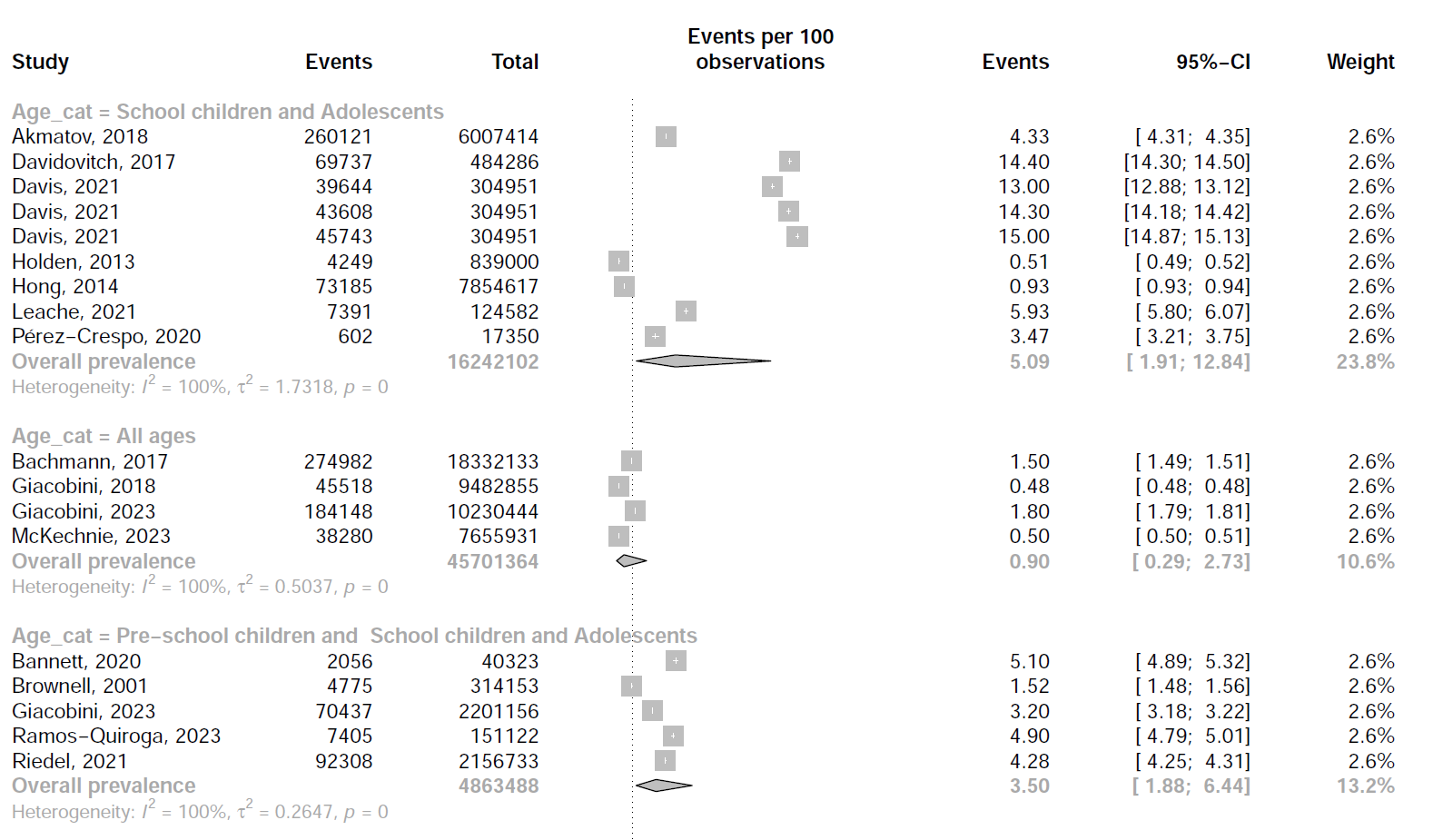


**Forest plot for Registry studies (Part 2)**


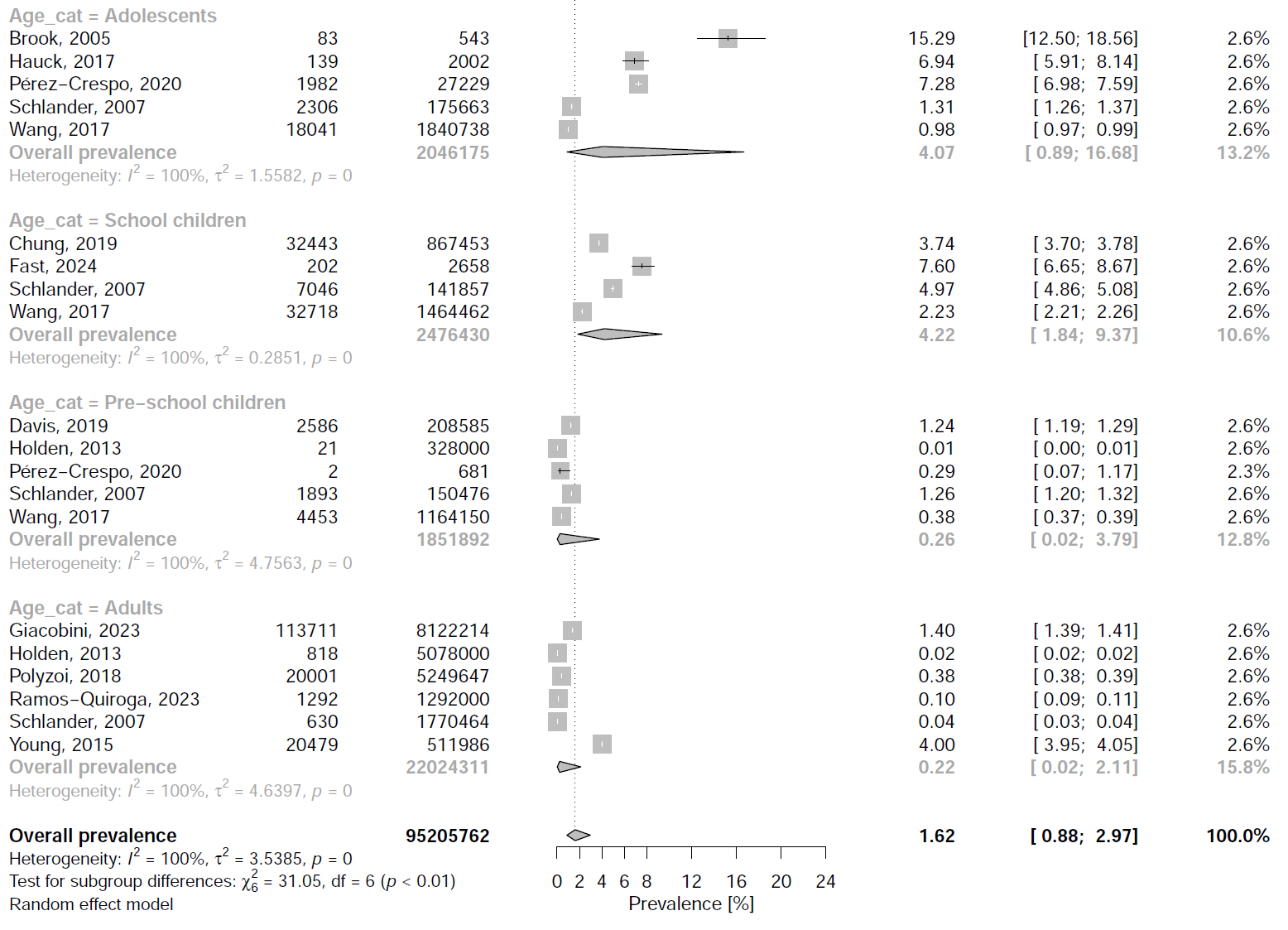

Supplement: Popit et al. supplementary material [file S0924933824017863sup001.docx]
